# 1-Methyl-tryptophan attenuates regulatory T cells differentiation due to the inhibition of estrogen-IDO1-MRC2 axis in endometriosis

**DOI:** 10.1038/cddis.2016.375

**Published:** 2016-12-01

**Authors:** Chunyan Wei, Jie Mei, Lingli Tang, Yukai Liu, Dajin Li, Mingqing Li, Xiaoyong Zhu

**Affiliations:** 1 Laboratory for Reproductive Immunology, Hospital and Institute of Obstetrics and Gynecology, Shanghai Medical School, Fudan University, Shanghai 200011, China; 2 Reproductive Medicine Center, Department of Obstetrics and Gynecology, Nanjing Drum Tower Hospital, The Affiliated Hospital of Nanjing University Medicine School, Nanjing 210000, China; 3 Shanghai Key Laboratory of Female Reproductive Endocrine Related Diseases, Shanghai 200011, China

## Abstract

Foxp3^+^ regulatory T (T_reg_) cells contribute to the local dysfunctional immune environment in endometriosis, an estrogen-dependent gynecological disease, which affects the function of ectopic endometrial tissue clearance by the immune system. The reason for the high percentage of peritoneal T_reg_ in endometriosis patients is unknown. Here, we show that the proportion of peritoneal T_reg_ cells increases as endometriosis progresses. To determine the probable mechanism, we established a naive T cell-macrophage-endometrial stromal cell (ESC) co-culture system to mimic the peritoneal cavity microenvironment. After adding 1-methyl-tryptophan (1-MT), a specific inhibitor of indoleamine 2,3-dioxygenase-1 (IDO1), to the co-culture system, we found that the differentiation of T_reg_ cells, mainly IL-10^+^ T_reg_ cells, decreased. Therefore, 1-MT-pretreated ESCs-educated T_reg_ cells performed impaired suppressive function. Moreover, estrogen promoted the differentiation of T_reg_ cells by elevating IDO1 expression in the ectopic lesion. Subsequently, we examined mannose receptor C, type 2 (MRC2), which is an up-stream molecule of IL-10, by bioinformatics analysis and real-time PCR validation. MRC2 expression in ectopic ESCs was notably lower than that in normal ESCs, which further negatively regulated the expression of IDO1 and Ki-67 in ESCs. Furthermore, MRC2 is required for T_reg_ differentiation in the ectopic lesion, especially that for CD4^high^ T_reg_. Therefore, MRC2-silenced ESCs-educated T_reg_ manifested a stronger suppressive function *in vitro*. Consistently, the percentage of T_reg_ increased when MRC2-shRNA was administered in the peritoneal cavity of endometriosis-disease mice model. Besides, 1-MT improved the condition of endometriosis, in terms of reducing the number and weight of total ectopic lesions *in vivo*. These results indicate that the estrogen-IDO1-MRC2 axis participates in the differentiation and function of T_reg_ and is involved in the development of endometriosis. Thus, blockage of IDO1 in the ectopic lesion, which does not influence physiological functions of estrogen, may be considered a potential therapy for endometriosis.

Under the influence of various factors, sloughed endometrial-like tissue in retrograde menstruation reaches the peritoneal cavity and adheres to endoabdominal structures to form ectopic lesions, resulting in dysmenorrhea, chronic pelvic pain and infertility, referred to as endometriosis (EMS).^1, 2^ At first, endometriosis was considered a benign, estrogen-dependent gynecological disease. However, it has been subsequently recognized as not only an endocrine disorder, but also a chronic inflammatory condition.

To date, three main aspects have been noted in the pathogenesis of endometriosis. Firstly, with regard to endometrial tissue, a lot of genes are differentially expressed in the ectopic endometrium compared with that in the eutopic and normal endometrium,^3, 4^ which may play pivotal roles in the development of endometriosis. We previously showed that the expression of indoleamine 2,3-dioxygenase-1 (IDO1), a rate-limiting enzyme that catalyzes the synthesis of tryptophan, is higher in ectopic endometrial stromal cells (ESCs) than that in normal ESCs. Additionally, IDO1 suppresses T-cell responses, promotes immune tolerance, and influences the differentiation of regulatory T (T_reg_) cells.^5^ We found that IDO1 promotes survival, proliferation, and invasion of ESCs via the JNK signaling pathway, but inhibits apoptosis of ESCs.^6^ The second aspect involved is abnormal endocrine function. High expression of mitochondrial cholesterol side-chain cleavage enzyme (CYP11A1) and hydroxysteroid (17 beta) dehydrogenase (HSD17B) in ectopic endometrial tissue increases local estrogen levels, which influences biological activities of ESCs,^7, 8^ leading to the development of endometriosis. Finally, the immunological aspect has been implicated, owing to evidence of immune tolerance in the endometriosis microenvironment, which is affected by changes in the proportion of T_reg_ cells,^9^ which in turn plays an important role in the maintenance of immune homeostasis to prevent potentially severe autoimmunity.^10, 11^ It has been reported that the percentage of T_reg_ cells in the peritoneal fluid of patients with endometriosis is higher than that in healthy women,^12^ and that Foxp3 expression by T_reg_ cells and T_reg_ function is increased in estrogen-treated mice.^13^ However, the exact mechanisms are unknown.

Considering the higher peritoneal T_reg_ cell percentage in EMS patients than that in healthy women, as well as findings that IDO1 controls T_reg_ cell function in response to inflammatory stimuli,^14, 15^ and higher expression of IDO1 in ectopic lesion regulates biological activities of ESCs in endometriosis,^6^ we reasoned that excessive estrogen may regulate IDO1 expression in the ectopic lesion to induce T_reg_ cell differentiation. In this study, we explored the origin of excessive T_reg_ cells in the peritoneal fluid of patients with endometriosis. To our knowledge, this is the first report showing that mannose receptor C, type 2 (MRC2), which is related to collagen turnover ^16^ and cancer prognosis,^17, 18^ plays a vital role in T_reg_ cell differentiation and function in endometriosis. Clinically, our findings may provide evidence that 1-methyl-tryptophan (1-MT) has potential applications in the treatment of endometriosis, retaining the physiological functions of estrogen.

## Results

### Peritoneal T_reg_ increases as endometriosis progresses

**Figure 1:**
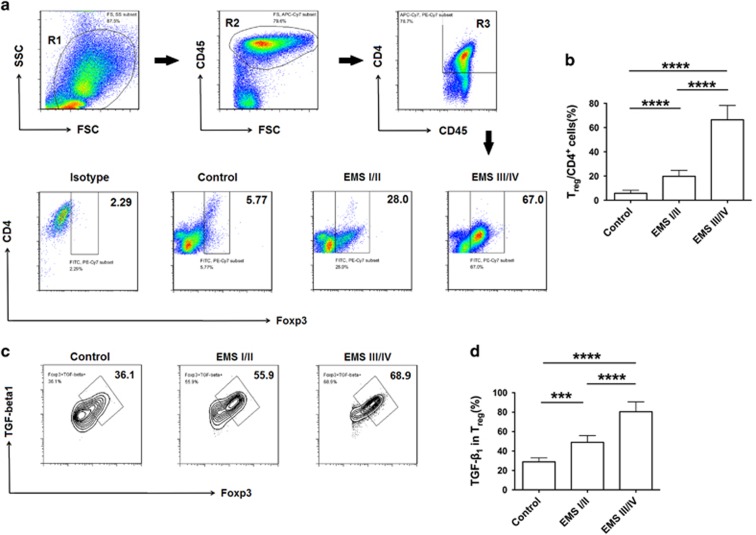
Percentage of T_reg_ cells in peritoneal fluid increases as endometriosis progresses. (**a**) Complete gating strategy for peritoneal T_reg_ cells. Gate R2 is inclusive of gate R1; cells of gate R2 represent CD45^+^ cells. Gate R3 is inclusive of gate R2; cells of gate R3 represent CD4^+^CD45^+^ cells. Peritoneal fluid from a patient with EMS stage III/IV is represented above. Flow cytometric analysis was used to determine the percentage of T_reg_ cells in peritoneal fluid of endometriosis patients in different stages. Numbers in quadrants indicate the percentage of cells. (**b**) Quantification of the percentage of T_reg_ cells in **a**. Values indicate mean±S.D., *n* (Control)=6, *n* (EMS stage I/II)=6, *n* (EMS stage III/IV)=6, ****P<0.0001, two-tailed, unpaired *t*-test. (**c**) Flow cytometric analysis was used to determine the expression of TGF- *β*_1_ in peritoneal T_reg_ cells of endometriosis patients in different stages. Numbers in quadrants indicate the percentage of cells. (**d**) Quantification of TGF-*β*_1_ expression of T_reg_ cells in **c**. Values indicate mean±S.D., *n* (Control)=6, *n* (EMS stage I/II)=6, *n* (EMS stage III/IV)=6, ***P<0.001, ****P<0.0001, two-tailed, unpaired *t*-test. EMS I/II, stage I and II of endometriosis; EMS III/IV, stage III and IV of endometriosis

The percentage of T_reg_ cells in peritoneal fluid is higher in patients with endometriosis than in healthy women.^12^ As shown in Figure 1a and b, the proportion of peritoneal T_reg_ cells in EMS stage III/IV patients was significantly higher than that in EMS stage I/II patients and healthy women. TGF-*β*_1_ expression in peritoneal T_reg_ cells also showed a similar trend (Figure 1c and d); however, IL-10 expression in T_reg_ cells did not (data not shown). These data demonstrated that the percentage of peritoneal T_reg_ cells increases with exacerbation of endometriosis.

### IDO1 participates in the differentiation of T_reg_ cells in endometriosis

**Figure 2:**
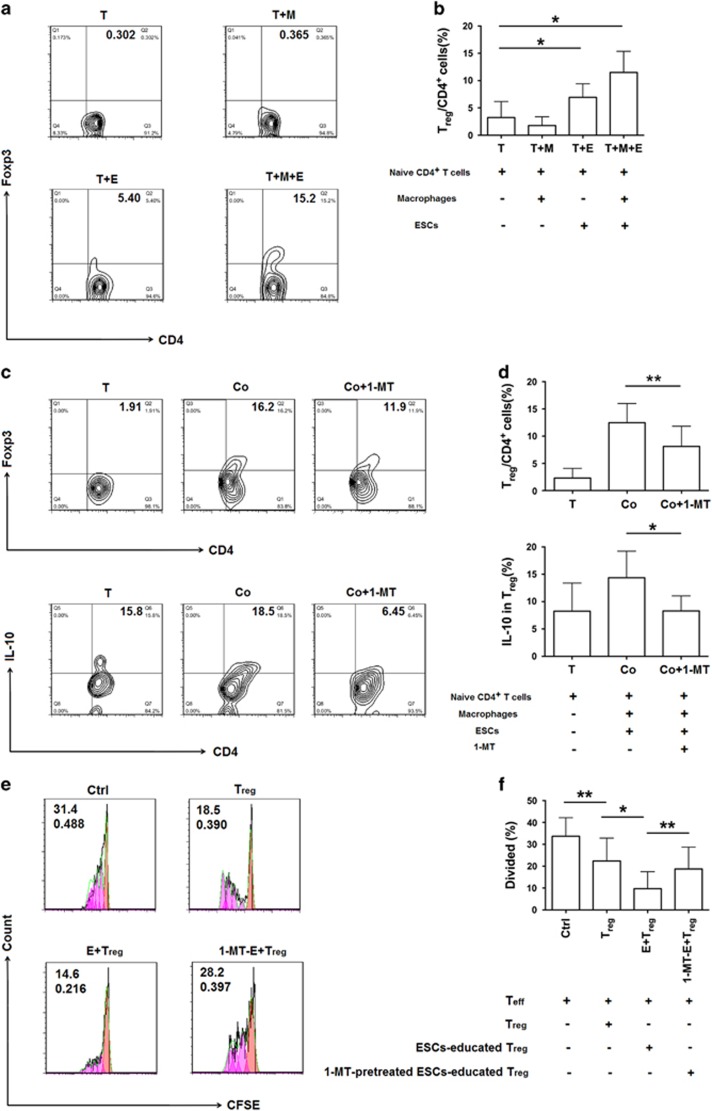

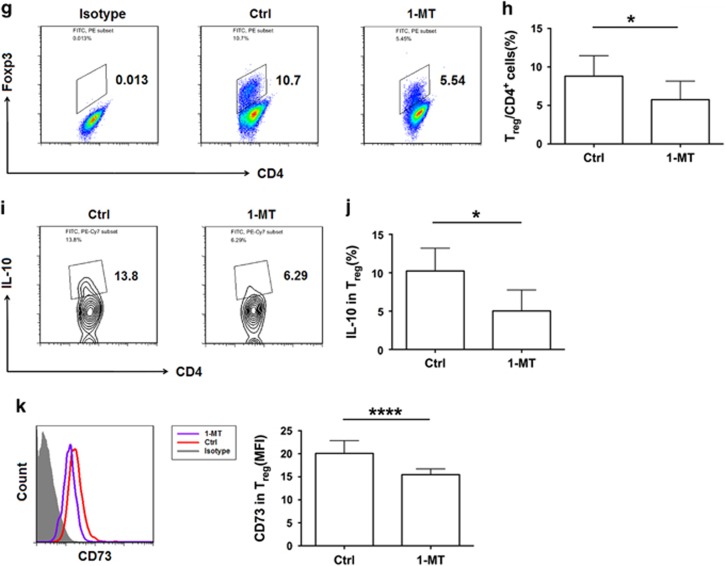
IDO1 is involved in the differentiation of T_reg_ in endometriosis. (**a**) Ectopic ESCs were incubated alone or co-cultured with positively sorted CD14^+^ cells from human peripheral blood, while negatively sorted naive CD4^+^ T cells from peripheral blood were cultured in 24-well plates that coated with 5 *μ*g/ml monoclonal anti-CD3 and 1 *μ*g/ml monoclonal anti-CD28, in the presence of 50 ng/ml recombinant human IL-2. After 48 hours, macrophages and naive CD4^+^ T cells were collected to establish co-culture systems. The percentage of CD4^+^Foxp3^+^ T cells in co-culture systems were determined by flow cytometric analysis after 5 days. Numbers in quadrants indicate the percentage of cells. (**b**) Quantification of T_reg_ cells in **a**. Values indicate mean±S.D., *n*=7, *P<0.05, two-tailed, paired *t*-test. (**c**) Flow cytometric analysis was used to determine the percentage of T_reg_ cells and the expression of IL-10 of T_reg_ cells in the co-culture system with or without 1-MT. Numbers in quadrants indicate the percentage of cells. (**d**) Quantification of the percentage of T_reg_ cells and the expression of IL-10 of T_reg_ cells in **c**. Values indicate mean±S.D., *n*=7, *P<0.05, **P<0.01, two-tailed, paired *t*-test. (**e**) Flow cytometric analysis was performed to determine the proliferation of T_eff_ cells (CFSE-labeled) from cultured with non-treated-, ESCs-educated-, 1-MT-pretreated ESCs-educated-T_reg_ cells or not. Numbers in quadrants indicate the percentage of divided cells and division index. Ctrl group contains T_eff_ cells alone; T_reg_ group contains T_eff_ cells and non-treated T_reg_ cells; E+T_reg_ group contains T_eff_ cells and ESCs-educated T_reg_ cells; 1-MT-E+T_reg_ group contains T_eff_ cells and 1-MT-pretreated ESCs-educated T_reg_ cells. E, ESCs. Peaks represent generations of cells. Salmon peak represents parental cells, magenta peaks represent daughter cells of T_eff_ cells. (**f**) Quantification of the divided percentage shown in (**e**). Values indicate mean±S.D., *n*=10, *P<0.05, **P<0.01, two-tailed, paired *t*-test. (**g**) Flow cytometric analysis was used to determine the percentage of CD4^+^Foxp3^+^ T cells in peritoneal fluid of mice with endometriosis in PBS- (Ctrl) and 1-MT- administered groups. Numbers in quadrants indicate the percentage of cells. (**h**) Quantification of the percentage of CD4^+^Foxp3^+^ T cells in (**g**). Values indicate mean±S.D., *n*=8, *P<0.05, two-tailed, unpaired *t*-test. (**i**) Flow cytometric analysis was used to determine the expression of IL-10 in peritoneal T_reg_ cells *in vivo*. Numbers in quadrants indicate the percentage of cells. (**j**) Quantification of IL-10 expression in T_reg_ cells shown in (**i**). Values indicate mean±S.D., *n*=8, *P<0.05, two-tailed, paired *t*-test. (**k**) MFI of the expression of CD73 in T_reg_ of peritoneal fluid from endometriosis-disease mice model in PBS (Ctrl) and 1-MT administration groups. Values are means±S.D., *n*=8, ****P<0.0001, two-tailed, unpaired *t*-test. T, naive CD4^+^ T cells; M, macrophages; E, ESCs; Co group in (**c** and **d**), the naive T cell-macrophage-endometrial stromal cell (ESC) co-culture system

To verify whether IDO1 influences the differentiation of T_reg_ cells in the ectopic lesion, we established co-culture systems to mimic the microenvironment of the peritoneal cavity (Supplementary Figure 1) and detected T_reg_ cell differentiation and expression of functional molecules in T_reg_ cells when treated with 1-MT or not. The highest percentage of T_reg_ was found in the naive T cell-macrophage-ESC co-culture system, compared with that in two other co-culture systems (Figure 2a and b). Therefore, we used this co-culture system for further analysis.

After adding 1-MT, a specific inhibitor of IDO1, to the co-culture system, the differentiation of T_reg_ cells decreased significantly, especially that of IL-10^+^ T_reg_ cells (Figure 2c and d), whereas, TGF-*β*_1_^+^ T_reg_ cell remained unchanged (data not shown). Moreover, 1-MT-pretreated ESCs-educated T_reg_ performed less suppressive function, as the divided percentage of CD4^+^CD25^−^ (T_eff_) cells was higher than that of E+T_reg_ group, while ESCs-educated T_reg_ cells owned a more suppressive function compared with non-pretreated-T_reg_ (Figure 2e and f). These results indicate that IDO1 is involved in the differentiation and suppressive function of T_reg_ cells in endometriosis. To investigate whether IDO1 participates in the differentiation of T_reg_ cells in endometriosis *in vivo*, we established an endometriosis-disease mouse model (Supplementary Figure 2). The results showed that 1-MT distinctly inhibited the percentage of T_reg_ cells in peritoneal fluid of mice (Figures 2g and h), especially IL-10 and CD 73 expression in peritoneal T_reg_ cells (Figures 2i and k), which were consistent with the *in vitro* results.

### IDO1 is up-regulated by estrogen in the ectopic lesion

**Figure 3:**
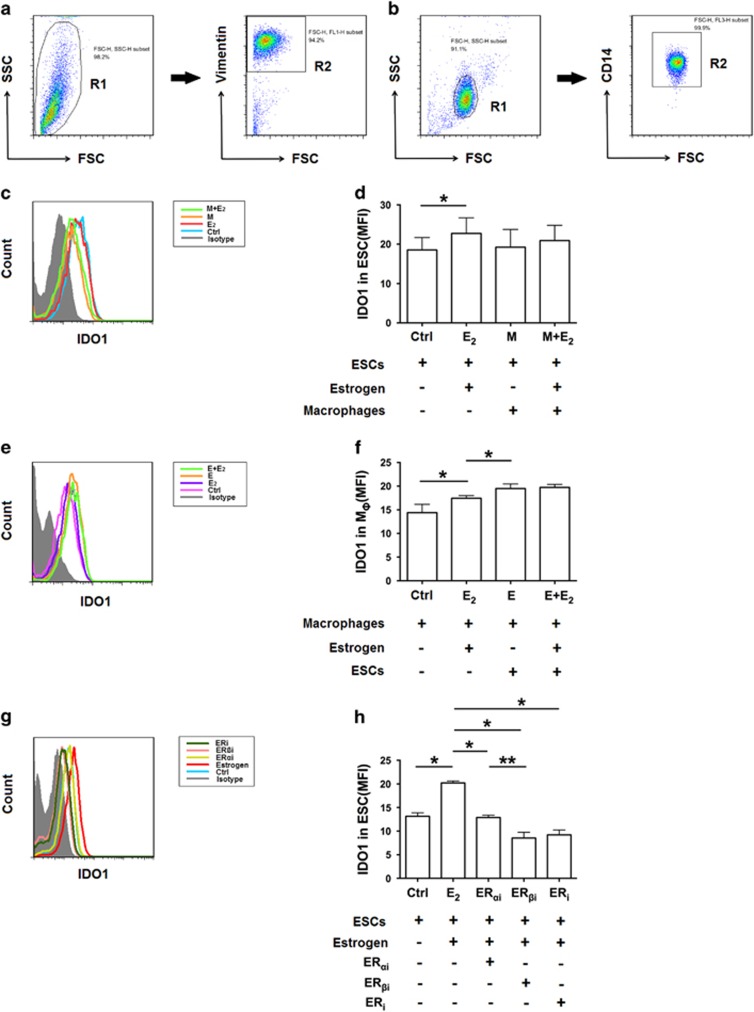
Expression of IDO1 is up-regulated by estrogen in the ectopic lesion. (**a**) Complete gating strategy of ectopic ESCs. Gate R2 is inclusive of gate R1; cells of gate R2 represent ESCs. (**b**) Complete gating strategy of monocytes. Gate R2 is inclusive of gate R1; cells of gate R2 represent CD14^+^ cells. (**c**) Flow cytometric analysis was used to determine the expression of IDO1 in ESCs (Ctrl), estrogen-treated ESCs (E_2_), monocyte-treated ESCs (M), and monocyte-treated ESCs in the presence of estrogen (M+E_2_). (**d**) MFI of the expression of IDO1 in groups shown in (**c**). Values indicate mean±S.D., *n*=5, *P<0.05, two-tailed, paired *t*-test. (**e**) Flow cytometric analysis was used to determine the expression of IDO1 in monocytes (Ctrl), estrogen-treated monocytes (E_2_), ESCs-treated monocytes (**e**), and ESCs-treated monocytes in the presence of estrogen (E+E_2_). (**f**) MFI of the expression of IDO1 in groups shown in (**e**). Values indicate mean±S.D., *n*=13, *P<0.05, two-tailed, paired *t*-test. (**g**) ESCs were pretreated with estrogen receptor-*α* inhibitor (ER_αi_), estrogen receptor-*β* inhibitor (ER_βi_), or estrogen receptors inhibitor (ER_i_) for 24 h, washed and then estrogen was added to each group, except control group. Control (Ctrl) group contained untreated ESCs. Flow cytometric analysis was used to determine the expression of IDO1 in ESCs from these groups. (**h**) MFI of the expression of IDO1 shown in (**g**). Values indicate mean±S.D., *n*=4, *P<0.05, **P<0.01, two-tailed, paired *t*-test

Patients with endometriosis show high local estrogen levels.^7^ Additionally, IDO1 expression in ectopic ESCs is higher than that in normal ESCs,^6^ leading us to consider that estrogen may regulate the expression of IDO1 in the ectopic lesion. We found that IDO1 expression in estrogen-conditioned ESCs and estrogen-conditioned macrophages were obviously higher than that in the control groups (Figures 3c–f). Besides, the effect of ESCs on up-regulating the expression of IDO1 in macrophages was more significant than that with estrogen alone (Figures 3e and f), which indicates a crosstalk between ESCs and macrophages that related to IDO1 expression.

To identify which sub-unit estrogen receptor (ER) that allows estrogen to promote the expression of IDO1 in ESCs, we blocked ER_*α*_, ER_*β*_, and ER respectively. We found that IDO1 expression in ESCs was down-regulated regardless of whether a single or both sub-unit ERs were blocked. This suggests that both sub-units of ER are involved in this activity, especially ER_*β*_ (Figures 3g and h). Although the percentage of T_reg_ cells in ectopic lesions of the estrogen receptor inhibitor (ER_i_) group showed little changes *in vivo* (data not shown), the percentage of TGF-*β*_1_^+^, IL-10^+^, CD73^+^, and CTLA-4^+^ T_reg_ cells decreased (Supplementary Figure 3).

### Estrogen induces the differentiation of T_reg_ cells via IDO1

**Figure 4:**
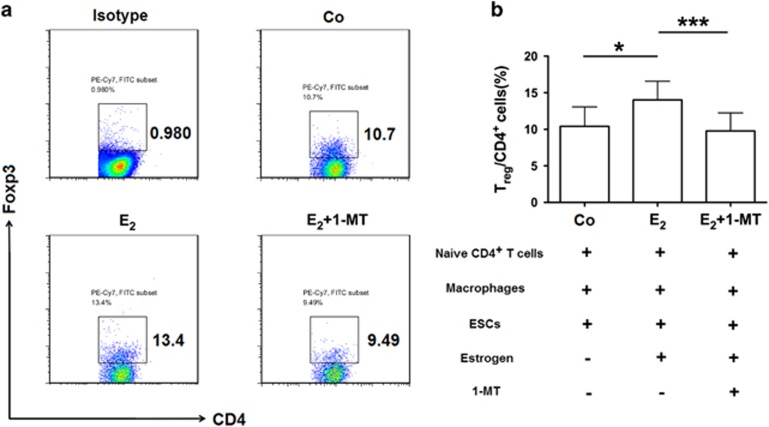
Estrogen promotes the differentiation of T_reg_ cells through elevated IDO1 levels in the ectopic lesion. (**a**) Flow cytometric analysis was used to determine the percentage of T_reg_ cells in the co-culture system obtained with estrogen, estrogen plus 1-MT, or neither. Numbers in quadrants indicate the percentage of cells. (**b**) Quantification of T_reg_ cells shown in **a**. Values indicate mean±S.D., *n*=12, *P<0.05, ***P<0.001, two-tailed, paired *t*-test. Co, the naive T cells-macrophage-endometrial stromal cell (ESC) co-culture system. E_2_, estrogen

Considering that endometriosis is an estrogen-dependent disease, and that estrogen enhances Foxp3 expression and T_reg_ cell function,^13^ we explored whether estrogen regulates the differentiation of T_reg_ cells in endometriosis. After adding estrogen to the co-culture system, the differentiation of T_reg_ cells notably increased (Figures 4a and b), which indicates that local high estrogen level participates in inducing the differentiation of T_reg_ cells in the peritoneal fluid of patients with endometriosis.

As mentioned above, estrogen promotes the expression of IDO1 in the ectopic lesion, and IDO1 influences the differentiation of T_reg_ cells,^5^ raising the possibility that estrogen may promote the differentiation of T_reg_ cells via up-regulation of IDO1 expression. Compared with that in the estrogen group, the differentiation of T_reg_ cells in estrogen plus 1-MT group was lower (Figures 4a and b), which manifested that 1-MT inhibits T_reg_ cell differentiation induced by estrogen.

### MRC2 is a downstream molecule of IDO1 and negatively regulates IDO1

**Figure 5:**
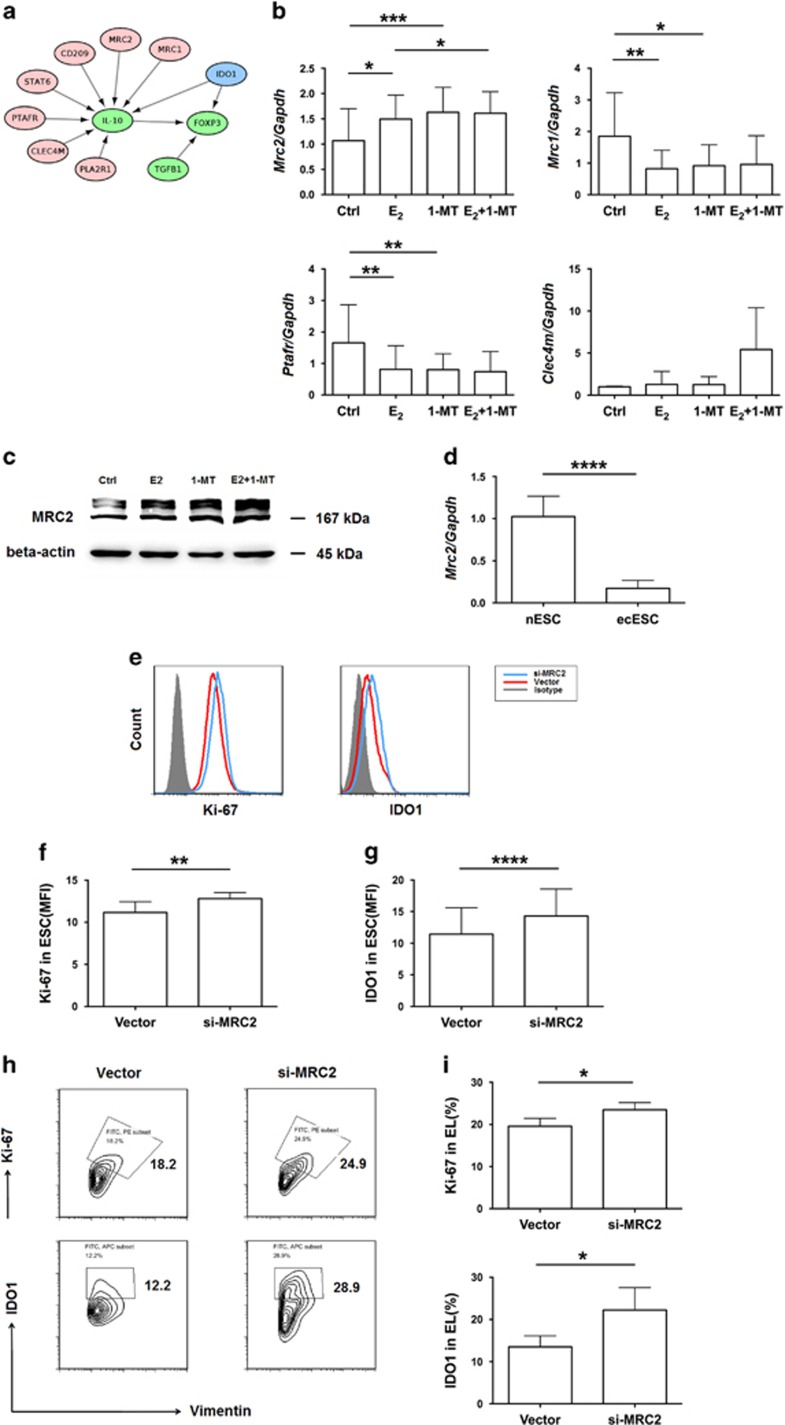
MRC2 is a downstream molecule of IDO1 and negatively regulates IDO1 expression. (**a**) Interactions of IDO1, IL-10, TGF-β_1_, and Foxp3. (**b**) mRNA expression of MRC2 (*n*=13), MRC1 (*n*=9), PTAFR (*n*=9), CLEC4M (*n*=6) in ESCs (Ctrl group), estrogen-treated ESCs (E_2_ group), 1-MT-treated ESCs (1-MT group), and estrogen plus 1-MT-treated ESCs (E_2_+1-MT group) was determined. Values indicate mean±S.D., *P<0.05, **P<0.01, ***P<0.001, two-tailed, paired *t*-test. (**c**) Protein expression of MRC2 shown in (**b**) analyzed by western blot. (**d**) mRNA expression of MRC2 in normal ESCs (nESC, *n*=9) and ectopic ESCs (ecESC, *n*=15). Values indicate mean±S.D., ****P<0.0001, two-tailed, unpaired *t*-test. (**e**) Flow cytometric analysis was used to determine the expression of IDO1 and Ki-67 in MRC2-silenced (si-MRC2) ESCs and ESCs of vector group. (**f, g**) MFI of the expression of IDO1 and Ki-67 shown in (**e**), *n*=8, **P<0.01, ****P<0.0001, two-tailed, paired *t*-test. (**h**) Flow cytometric analysis was used to determine the expression of Ki-67 and IDO1 in ectopic lesions of vector- and MRC2 shRNA-administered groups *in vivo*. Numbers in quadrants indicate the percentage of cells. (**i**) Quantification of the expression of Ki-67 and IDO1 in ectopic lesions in (**h**). Values indicate mean±S.D., *n*=6, *P<0.05, two-tailed, unpaired *t*-test

As outlined above, IDO1 influences the differentiation of T_reg_ cells, especially IL-10^+^ T_reg_ cells. To identify factors that possibly connect IDO1 and IL-10, and are involved in interactions of IL10, IDO1, Foxp3, and TGF-*β*, we performed a search of the KEGG database as part of the bioinformatics analysis. We found that molecules up-stream of IL-10 include C-type lectin domain family 4 member M (CLEC4M), mannose receptor C, type 1 (MRC1), mannose receptor C, type 2 (MRC2), STAT6, phospholipase A2 receptor1 (PLA2R1), platelet-activating factor receptor (PTAFR), and CD209 (Figure 5a). After treating ESCs with estrogen, 1-MT, or estrogen plus 1-MT, only MRC2 expression increased in the estrogen-treated group compared with that in the control group (Figure 5b), which was similar to our previous result where estrogen up-regulated IDO1 expression in ESCs (Figures 3c and d). Thus, the results demonstrate that 1-MT also promotes the expression of MRC2 in ESCs to a greater extent than estrogen (Figures 5b and c), which means that MRC2 is downstream to IDO1 and estrogen.

MRC2 is a constitutively recycling endocytic receptor belonging to the mannose receptor family.^19^ The expression of MRC2 in ectopic ESCs was significantly lower than that in normal ESCs (Figure 5d). Combined with evidence that the expression of IDO1 in ectopic ESCs is higher than that in normal ESCs,^6^ and 1-MT up-regulates the expression of MRC2, we reasoned that high levels of IDO1 might lead to low expression of MRC2 in ectopic ESCs. Besides, after silencing MRC2 in ESCs, the expression of IDO1 increased (Figures 5e and g), which indicates a negative feedback between MRC2 and IDO1. Therefore, the level of Ki-67 in MRC2-silenced ESCs increased compared with that in the vector group (Figures 5e and f), which is consistent with the notion that ectopic ESCs have a stronger ability for proliferation.^6^ Similarly, the expression of Ki-67 and IDO1 also increased in ectopic lesions after MRC2 shRNA was intraperitoneal injected to the peritoneal cavity of endometriosis mice model *in vivo* (Figures 5h and i).

### MRC2 is required for the differentiation of T_reg_ cells in endometriosis

**Figure 6:**
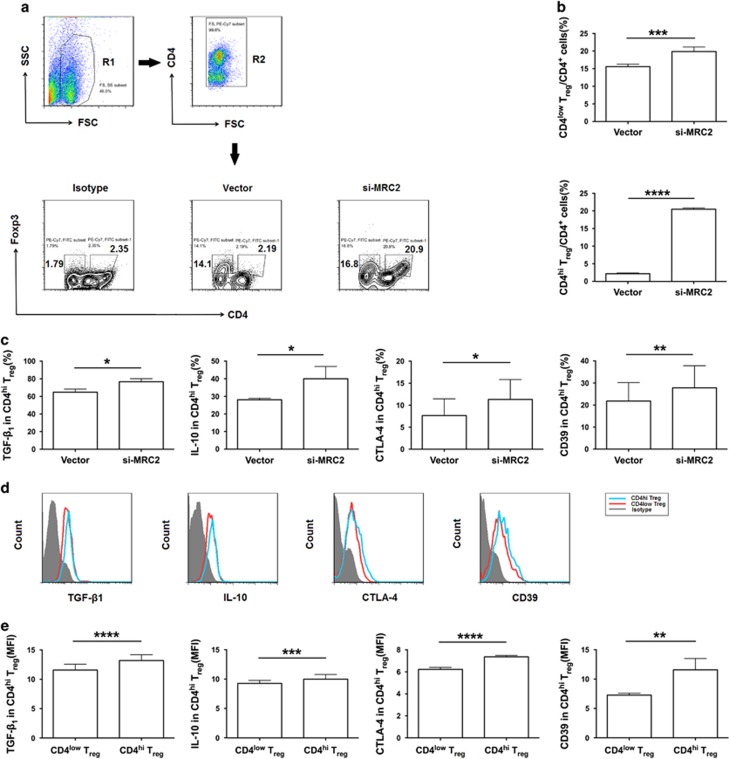

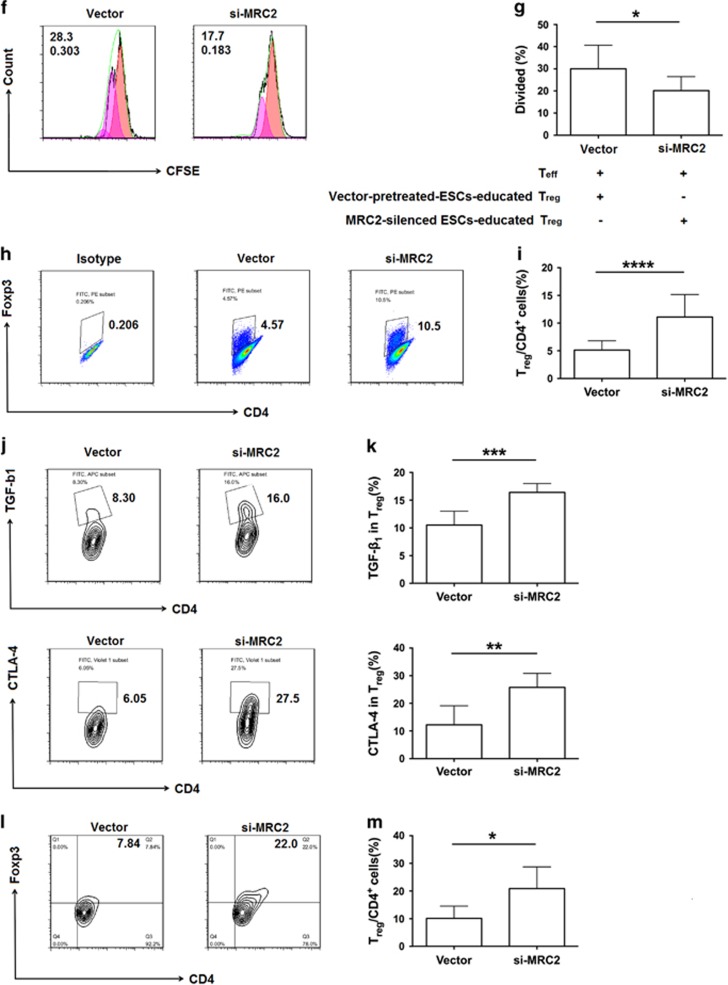
MRC2 is required for the differentiation and immunosuppressive function of T_reg_ cells in endometriosis. (**a**) Complete gating strategy of T_reg_ cells from the co-culture system. Gate R2 is inclusive of gate R1; cells of gate R2 represent CD4^+^ cells. Flow cytometric analyses CD4^+^Foxp3^+^ T cells in the co-culture system contained MRC2-silenced ESCs or vector-treated ESCs. Numbers in quadrants indicate the percentage of T_reg_ cells. (**b**) Quantification of CD4^low^ and CD4^high^ T_reg_ cells in **a**. Values indicate mean±S.D., *n*=6, ***P<0.001, ****P<0.0001, two-tailed, paired *t*-test. (**c**) Expression of TGF-β_1_ (*n*=5), IL-10 (*n*=4), CTLA-4 (*n*=7), and CD39 (*n*=6) in CD^high^ T_reg_ cells shown in (**a**). Values indicate mean±S.D., *P<0.05, **P<0.01, two-tailed, paired *t*-test. (**d**) Flow cytometric analysis was performed to determine the expression of TGF-β_1_, IL-10, CTLA-4, and CD39 in CD4^high^ T_reg_ cells of si-MRC2 group shown in (**a**). (**e**) MFI of TGF-β_1_ (*n*=6), IL-10 (*n*=8), CTLA-4 (*n*=7) and CD39 (*n*=7) in CD4^high^ T_reg_ cells of si-MRC2 group shown in **a**. Values indicate mean±S.D., **P<0.01, ***P<0.001, ****P<0.0001, two-tailed, paired *t*-test. (**f**) Flow cytometric analysis was performed to determine the proliferation of T_eff_ cells (CFSE-labeled) from cultured with vector-pretreated-ESCs-educated-, or MRC2-silenced ESCs-educated T_reg_ cells. Numbers in quadrants indicate the percentage of divided cells and division index. Vector group contains T_eff_ cells and vector-pretreated-ESCs-educated T_reg_ cells; si-MRC2 group contains T_eff_ cells and MRC2-silenced ESCs-educated T_reg_ cells. Peaks represent generations of cells. Salmon peak represents parental cells, magenta peaks represent daughter cells of T_eff_ cells. (**g**) Quantification of the divided percentage of T_eff_ cells shown in (**f**). Values indicate mean±S.D., *n*=6, *P<0.05, two-tailed, paired *t*-test. (**h**) Flow cytometric analyses peritoneal CD4^+^Foxp3^+^ T cells *in vivo* from vector-administered or MRC2 shRNA-administered group. Numbers in quadrants indicate the percentage of T_reg_ cells. (**i**) Flow cytometric analysis was performed to determine the percentage of peritoneal CD4^+^Foxp3^+^ T cells of vector group or si-MRC2 group *in vivo*. Values indicate mean±S.D., *n*=13, ****P<0.0001, two-tailed, unpaired *t*-test. (**j**) Flow cytometric analyses expression of TGF-β_1_ and CTLA-4 of peritoneal CD4^+^Foxp3^+^ T cells *in vivo* from vector-administered or MRC2 shRNA-administered group. Numbers in quadrants indicate the percentage of cells. (**k**) Expression of TGF-β_1_ and CTLA-4 in peritoneal T_reg_ cells *in vivo* shown in (**j**). Values indicate mean±S.D., *n*=7, ***P<0.001, two-tailed, unpaired *t*-test. (**l**) Flow cytometric analyses the percentage of CD4^+^Foxp3^+^ T cells in ectopic lesions *in vivo* from vector-administered or MRC2 shRNA-administered group. Numbers in quadrants indicate the percentage of cells. (**m**) Quantification of T_reg_ cells shown in (**l**). Values indicate mean±S.D., *n*=6, *P<0.05, two-tailed, unpaired *t*-test. si-MRC2, silenced-MRC2; CD4^hi^ T_reg_, CD4^high^ T_reg_

According to the findings above, MRC2 is downstream to IDO1, and IDO1 is involved in the differentiation of T_reg_ in ectopic lesion, hinting the possibility that MRC2 may participate in the activity that IDO1 regulates the differentiation of T_reg_ in endometriosis. When MRC2-silenced ESCs were co-cultured with naive CD4^+^ T cells and monocytes-derived macrophages, the percentage of CD4^low^ T_reg_ and CD4^high^ T_reg_ cells were higher in the MRC2-silenced group than that in the vector group, especially CD4^high^ T_reg_ cells (Figures 6a and b). Moreover, CD4^high^ T_reg_ cells from the MRC2-silenced group showed a more immunosuppressive phenotype, with higher expression of TGF-*β*1, IL-10, CD39 and CTLA-4, than that in CD4^high^ T_reg_ cells in the vector group and CD4^low^ T_reg_ cells in MRC2-silenced group (Figures 6c and e). Furthermore, MRC2-silenced ESCs-educated T_reg_ performed stronger suppressive function, as divided percentage of T_eff_ cells was lower than that of vector group, which indicated that MRC2 is involved in T_reg_ suppressive function (Figures 6f and g). *In vivo*, at two weeks after administrating MRC2-shRNA via the peritoneal cavity of the endometriosis-disease mouse model, the percentage of peritoneal T_reg_ cells and T_reg_ cells in ectopic lesions were significantly higher than that in the vector group (Figures 6h, i, l and m), and mainly comprised TGF-*β*1^+^ and CTLA-4^+^ T_reg_ cells in the peritoneal fluid (Figures 6j and k). These results demonstrate that MRC2 is not only responsible for the differentiation of T_reg_ cells, mainly that of CD4^high^ T_reg_ cells; but also promotes T_reg_ immunosuppressive function in endometriosis, which may exacerbate the development of endometriosis.

### 1-MT reverses the development of endometriosis *in vivo*

**Figure 7:**
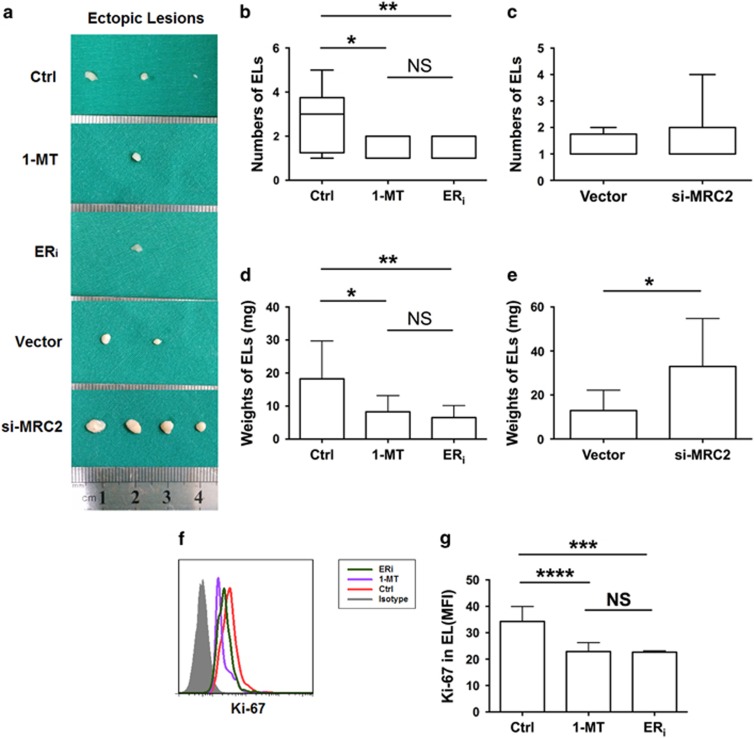
1-MT reverses the development of endometriosis *in vivo*. (**a)** Represent of ectopic lesions from PBS- (Ctrl), 1-MT-, ERi-, Vector-, or MRC2 sh-RNA-administered group. (**b** and **c**) Numbers of total ectopic lesions in mice administered PBS (*n*=8), 1-MT (*n*=9), estrogen receptor inhibitor (*n*=10), vector (*n*=8), and MRC2-shRNA (*n*=11). Values indicate mean±S.D., *P<0.05, **P<0.01, two-tailed, unpaired *t*-test. (**d, e**) Weights of total ectopic lesions from PBS (*n*=8), 1-MT (*n*=9), estrogen receptor inhibitor (*n*=10), vector (*n*=8) and MRC2-shRNA (*n*=11) administered groups *in vivo*. Values indicate mean±S.D., *P<0.05, two-tailed, unpaired *t*-test. (**f** and **g**) Flow cytometric analysis was used to determine Ki-67 expression (MFI) in ectopic lesions shown in PBS (*n*=8), 1-MT (*n*=9), estrogen receptor inhibitor (*n*=10) administration group *in vivo*. Values indicate mean±S.D., ***P<0.001, ****P<0.0001, two-tailed, unpaired *t*-test. ER_i_, estrogen receptor inhibitor; si-MRC2, silenced-MRC2; Ctrl group, PBS administration group

To determine whether 1-MT improves the condition of endometriosis *in vivo*, we measured the total number and weight of ectopic lesions and found that both parameters decreased notably in the 1-MT administered group compared with that in the control group, as similar as ERi administered group (Figures 7a, b and d). In the MRC2-shRNA administered group, the total number of ectopic lesions did not significantly increased (Figure 7c), but total weight of ectopic lesions was obviously higher than that in the vector group (Figure 7e), which indicates that MRC2 is responsible for ectopic lesion growth while not for the spread of the implant. Moreover, 1-MT notably restrained the proliferation of ectopic lesions *in vivo*, in terms of lower expression of Ki-67 compared with that in the control group, as seen with the ER_i_ group (Figures 7f and g). These results suggest that 1-MT reverses the condition of endometriosis *in vivo*.

## Discussion

Although endometriosis is an estrogen-dependent disease, it is not enough to determine the pathogenesis of endometriosis from the endocrine perspective alone. Multiple factors participate in the development and maintenance of endometriosis, including immunological dysfunction, genetic susceptibility, psychological factors, and environment factors. Among these, the immunological aspect of endometriosis has recently been extensively studied. Up to 90% of women of reproductive age exhibit retrograde menstruation, whereas only 6–10% of these women develop endometriosis.^20^ This indicates that a microenvironment of immune tolerance is formed within the ectopic lesion in patients with endometriosis, where the function of menstrual debris clearance by the local immune responses is ineffectively.^21^

Several types of immune cells are involved in the formation of the local immune tolerance environment in endometriosis, such as T_reg_ cells, macrophages, and natural-killer (NK) cells. Disturbance of T_reg_ cells, which are responsible for self-tolerance, maintenance of immune homeostasis, and immunosuppressive functions,^22^ aggravates the condition of endometriosis. In this study, we have demonstrated not only that the percentage of T_reg_ cells in the peritoneal fluid of endometriosis patients higher than that in healthy women, which is consistent with results of previous published studies,^23^ but also that the augmentation of T_reg_ cell percentage, especially that of TGF-*β*1^+^ T_reg_ cells, occurs in parallel with endometriosis exacerbation. This suggests that the detection of peritoneal T_reg_ cells may be used as an indicator to assess the severity of endometriosis. However, the origin of excessive T_reg_ cells in the peritoneal fluid of patients with endometriosis is yet unclear. Three assumptions may elucidate this clinic phenomenon. One is homing of peripheral T_reg_ cells to the peritoneal fluid as the percentage of T_reg_ cells decreases in the peripheral blood of EMS patients.^23^ The second is that local naive T cells differentiate into T_reg_ cells under the influence of various factors involved; and the third is self-proliferation of local T_reg_ cells. In fact, all of these assumptions may be involved in the increase of peritoneal T_reg_ cell percentage in endometriosis.

IDO1 is essential for the generation and function of T_reg_ cells.^14, 24, 25^ Initially, IDO1 was thought as the first line of host defense against infectious pathogens, as it causes tryptophan shortage, which in turn restricts mammalian cell growth,^26^ especially that of T cells.^27^ Subsequent studies revealed that IDO1 has multiple immunological functions, such as suppressing T-cell responses, regulating functions of T_reg_ cells,^14^ and promoting immune tolerance.^15^ Evidence of higher IDO1 expression in ectopic ESCs than in normal ESCs^6^ and high percentage of peritoneal T_reg_ cells in endometriosis^23^ demonstrates that IDO1 may play an important role in inducing the differentiation of T_reg_ cells in the ectopic lesion, which is a probable reason for excessive T_reg_ cells in the peritoneal fluid of patients with endometriosis.

In this study, we established a naive T cell-macrophage-ESC co-culture system to mimic the local microenvironment of the peritoneal cavity. The results of our study show that IDO1 participates in the differentiation of T_reg_ cells in the ectopic lesion, evidenced by inhibition of T_reg_ cell differentiation, especially that of IL-10^+^ T_reg_ cells, by 1-MT. In addition, 1-MT-pretreated ESCs-educated T_reg_ cells suppressed the proliferation of T_eff_ cells less effectively compared with non-treated ESCs-educated T_reg_ cells, which indicates that 1-MT is involved in T_reg_ cell suppressive function. Moreover, estrogen promoted the expression of IDO1 in both ESCs and macrophages, which, on a side-note, explains that ectopic ESCs have high expression of IDO1 in endometriosis. Therein, the expression of IDO1 in ESCs-educated macrophages was notably higher than that in both estrogen-treated and untreated monocytes-derived macrophages. This demonstrates a crosstalk between ESCs and macrophages, involving IDO1. Besides, estrogen was found to induce T_reg_ cell differentiation in the ectopic lesion and IDO1 was involved in this process, wherein, 1-MT down-regulated T_reg_ cell differentiation induced by estrogen. These results indicate that estrogen regulates the expression of IDO1 in the ectopic lesion to induce the T_reg_ cell differentiation.

Based on the finding that 1-MT mainly influences the differentiation of IL-10^+^ T_reg_ cells; we performed a search of the KEGG database to identify possible molecules that connect IDO1 and IL-10. The results show that MRC2, which is an upstream molecule of IL-10, is essential for the differentiation and function of T_reg_ cells. Most researches on MRC2 to date have focused on its role in the development of cancer, as it can promote breast tumor growth,^17^ co-operate with the matrix metalloproteinase to remodel of extracellular matrix that attenuate renal fibrosis,^28^ and predict prognosis of hepatocellular carcinoma^18^ and prostate cancer.^29^ Besides, MRC2 is also closely related to collagen turnover.^30^ In our study, we found that both estrogen and 1-MT promoted the expression of MRC2 in ESCs, which indicates that MRC2 is downstream to estrogen and IDO1. Therein, the regulatory ability of 1-MT was stronger than that of estrogen. Combined with the low expression of MRC2 by ectopic ESCs and MRC2 expression promoted by estrogen, we reasoned that other factors might be responsible for decreased MRC2 expression by ESCs. As shown in Figures 5b and c, we observed that 1-MT promoted the expression of MRC2 by ESCs to a greater extent than estrogen, whereas, decreased IDO1 levels caused an increase in MRC2 expression. Therefore, it is a possibility that increased IDO1 levels down-regulate the expression of MRC2 by ESCs. In addition, negative feedback was noted between IDO1 and MRC2, evidenced by increase in IDO1 expression when MRC2 in ESCs was silenced. Meanwhile, MRC2 negatively regulated proliferation of ESCs as the expression of Ki-67 increased in si-MRC2 ESCs compared with vector group. To explore the possibility that MRC2 influences the differentiation of T_reg_ cells, we co-cultured MRC2-silenced ESC with macrophages and naive CD4^+^ T cells. Compared with that in the vector group, the percentage of T_reg_ cells increased in the MRC2-silenced group, especially CD4^high^ T_reg_ cells, which demonstrate that MRC2 plays a key role in the differentiation of T_reg_ cells in endometriosis. Therefore, MRC2-silenced ESCs-educated T_reg_ cells acquired stronger suppressive function than vector-pretreated ESCs-educated T_reg_ cells, which indicates that MRC2 negatively regulates T_reg_ cell suppressive function. To our knowledge, this is the first report demonstrating the immunological functions of MRC2 as an important regulator of T_reg_ cell differentiation and function, and showing that estrogen and IDO1 are up-stream to MRC2. These results illustrate that estrogen-IDO1-MRC2 axis is involved in the differentiation of T_reg_ cells in endometriosis.

Consistent with the *in vitro* results above, 1-MT dramatically reversed the condition of endometriosis *in vivo*, irrespective of total number or weight of ectopic lesions, expression of Ki-67 in total ectopic lesions, or the percentage of peritoneal T_reg_ cells. These data suggest that locally applied 1-MT may clinically relieve sufferings of patients with endometriosis. Besides, MRC2 significantly induced the differentiation of T_reg_ cells, increased weight of total ectopic lesions, and promoted the expression of Ki-67 in ectopic lesions *in vivo*, which is consistent with the *in vitro* results, suggesting that MRC2 is involved in the growth of ectopic lesions and T_reg_ differentiation in endometriosis (Supplementary Figure 4).

Although endometriosis is a benign gynecological disease, its biological activities are similar to that of cancer, including metastasis, plantation, angiogenesis, immune tolerance, and recurrence.^31, 32^ In the present study, we focused on IDO1, MRC2, and T_reg_ cells, which have been reported in previous cancer studies,^17, 33–35^ to identify the endocrine-immune mechanism for the high percentage of peritoneal T_reg_ cells in patients with endometriosis. The results revealed that the percentage of T_reg_ cells in the peritoneal fluid increases as endometriosis progresses. Therein, we found that the lower expression of MRC2 in ectopic ESCs significantly promoted the differentiation and function of T_reg_ cells. This is a novel function of MRC2, identified in this study. Future work would involve identifying probable mechanisms by which MRC2 influences the differentiation of T_reg_ cells, including the transcription factors involved in this process and interactions of MRC2 and T_reg_ functional molecules. Moreover, the possibility that MRC2 influences the biological activities of ESCs in endometriosis remains to be explored. Common treatment options for endometriosis, including progestogens, ovulation induction, GnRH analog and surgery, however, typically do not provide long-term relief.^36^ Pharmacotherapy for endometriosis affects the physiological functions of estrogen and this limits its long-term use. In addition, high recurrence rate renders endometriosis clinically intractable. Therefore, to treat endometriosis, it is essential to identify possible targets that do not influence the physiological functions of estrogen. The *in vivo* results from this study showed that 1-MT, a specific inhibitor of IDO1, can notably improve the condition of endometriosis, as similar as ER inhibitor, which provide evidences that blocking IDO1 in ectopic lesions may be a novel treatment option for endometriosis, not only by virtue of its effects on biological activities of ESCs, but also on local immune-tolerance environment effected by inhibition of the differentiation and functions of T_reg_ cells. To date, 1-MT is undergoing clinical phase II and I trials for application in cancer immunotherapy.^15^ Intrauterine devices containing 1-MT can be considered for the treatment of endometriosis. These data indicate that endometriosis is more than a disease involving endocrine disorders, and also involves immunological factors. Moreover, it should be taken into consideration that assessment of the local immunological status of patients with endometriosis is essential for the treatment choice to be made.

## Materials and Methods

### Ethics statement

The human ethics committee of Gynecology and Obstetrics Hospital, Shanghai Medical School, Fudan University, People's Republic of China approved this study. Written informed consent was obtained from each patient enrolled.

### Patients

Endometriotic tissues were acquired from premenopausal patients who had undergone laparoscopic surgery for the treatment of ovary endometriosis cysts (*n*=55; aged 22–46 years) diagnosed by pathological examination. Control endometrial tissues were obtained from premenopausal patients who had undergone panhysterectomy surgery for multiple myoma (*n*=15; aged 46–52 years). Peritoneal fluid was aspirated from the cul de sac at the beginning of the laparoscopic procedure under general anesthesia, obtained from patients with ovary endometriosis (*n*=16; aged 25–46 years), or patients who had laparoscopic surgery for uterine fibroid without evidence of endometriosis (*n*=12; aged 29–41 years) at the Obstetrics and Gynecology Hospital of Fudan University, between August 2014 and September 2016. Samples of peritoneal fluid contaminated by blood were excluded from the study. None of women had received hormonal therapy within 6 months prior to tissue collection. The stage of endometriosis was diagnosed according to the revised American Society for Reproductive Medicine staging (1997). Peripheral blood samples (5–15 ml) were obtained from healthy volunteers (*n*=35; aged 25–38 years old). All samples were collected under sterile conditions.

### Peritoneal T_reg_ detected by flow cytometry

All collected peritoneal fluid samples were centrifuged at 1600 rpm, at 4 °C for 9 min, then supernatant was discarded, and cells were fixed with 4% par formaldehyde (Sheng Gong, Shanghai, China) for 35 min, at 4 °C in the dark. Then cells were washed twice with phosphate-buffered saline (PBS; Hyclone, Logan, UT, USA). After centrifugation and removal of the supernatant, the fixed cells were resuspended in Foxp3 Perm Buffer (10 × , Biolegend, San Diego, USA) according to the manufacturer protocol. Finally, these cells were labeled with flow cytometry antibodies according to the manufacturer protocol and detected by flow cytometry.

### ESCs isolation

Endometrial stromal cells (ESCs) were purified as previously described.^37^ Endometriotic tissues were cut into<1mm-thick sections and digested with Dulbecco's Modified Eagle Medium (DMEM)/F12 (Hyclone, Logan, UT, USA) containing collagenase type IV (0.1% Sigma, San Francisco, CA, USA) with constant agitation for 40 min at 37 °C. The resulting suspension was then filtered through 100- to 70-*μ*m nylon strainers (Becton Dickinson, Franklin Lakes, NJ, USA). After the filtrate was centrifuged at 1600 r.p.m. for 9 min at 4 °C, the supernatant was discarded. Finally, ESCs were resuspended in DMEM/F-12 containing 10% fetal bovine serum (FBS; Hyclone) in the presence of 100 U/ml penicillin and 100 mg/ml streptomycin, and placed in culture flasks at 37 °C under 5% CO_2_. The culture medium was replaced with fresh medium every 3 days.

### Generation of human naive CD4^+^ T cells and monocytes

Peripheral blood mononuclear cells (PBMCs) were isolated by Lymphoprep (Stemcell Technologies Inc., Vancouver, BC, Canada) density gradient centrifugation. CD14^+^ cells were obtained by positively selection using CD14^+^ cells micro-magnetic beads according to the manufacturer instructions (Miltenyi Biotec, Bergisch Gladbach, Germany). Naïve CD4^+^ T cells were obtained by negatively selection from the remaining immune cells according to the manufacturer instructions (Miltenyi Biotec). The purity of CD14^+^ cells was confirmed by flow cytometry, using PE/Cy7 anti-human CD14 monoclonal antibody (mAb) (Biolegend, San Diego, USA, clone: HCD14), was found to be>95%.

### Cell co-culture systems

Ectopic ESCs were cultured in 24-well plates (Corning, Steuben County, NY, USA) at a density of 1 × 10^5^ cells/well. The co-culture systems were established by incubating 2 × 10^5^ monocytes with ESCs or alone, adding of estrogen (10^−8^ M; Sigma), 1-MT (0.05 mM; Sigma), or estrogen (10^−8^ M; Sigma) plus 1-MT (0.05 mM; Sigma). Meanwhile, naive CD4^+^ T cells were cultured in 24-well plates that coated with monoclonal anti-CD3 (5 *μ*g/ml; Biolegend) and monoclonal anti-CD28 (1 *μ*g/ml; Biolegend), in the presence of recombinant human IL-2 (50 ng/ml; Biolegend). The monocytes-derived macrophages and 2 × 10^5^ naive CD4^+^ T cells were collected to co-culture with ESCs in 1 ml medium/well for 5 days (Supplementary Figure 1).

### T_reg_ cell suppression assay

Peripheral blood mononuclear cells (PBMCs) were isolated by Lymphoprep (Stemcell Technologies Inc.) density gradient centrifugation. CD4^+^CD25^+^ T cells were obtained by positively selection using CD4^+^CD25^+^ T cells (Regulatory T cells) micro-magnetic beads according to the manufacturer instructions (Miltenyi Biotec). CD4^+^CD25^−^ cells were obtained by negatively selection according to the manufacturer instructions (Miltenyi Biotec). CD4^+^CD25^+^ T cells were cultured in 96-well plates that coated with monoclonal anti-human CD3 (5 *μ*g/ml; Biolegend, clone: OKT3) and monoclonal anti-human CD28 (1 *μ*g/ml; Biolegend, clone: CD28.2), in the presence of recombinant human IL-2 (50 ng/ml; Biolegend), while CD4^+^CD25^−^ cells were cultured in 24-well plates that coated with monoclonal anti-CD3 (5 *μ*g/ml; Biolegend) and monoclonal anti-CD28 (1 *μ*g/ml; Biolegend). T_reg_ cells were collected to culture with non-treated ESCs, 1-MT-pretreated ESCs, vector-pretreated ESCs, and MRC2-silenced ESCs or not for 48 h after T_reg_ cells proliferation for two weeks. After that, T_reg_ cells from these groups respectively cultured with paired CFSE-labeled CD4^+^CD25^−^ T cells (T_eff_) for 48 h and then detected by flow cytometry.

### Antibodies and flow cytometry

Fluorochrome-conjugated antibodies the following human antigens were used for flow cytometry analysis: Alexa Fluor 488 anti-human Foxp3 mAb (Biolegend, clone: 206D), PE anti-human LAP (TGF-*β*1) mAb (Biolegend, clone: TW4-6H10), PE/Cy7 anti-human CD4 mAb (Biolegend, clone: RPA-T4), APC anti-human IL-10 mAb (Biolegend, clone: JES3-9D7), PerCP/Cy5.5 anti-human CD73 (Ecto-5'-nucleotidase) mAb (Biolegend, clone: AD2), Brilliant Violet 421^TM^ anti-human CD152 (CTLA-4) mAb (BD Biosciences, clone: BNI3), Brilliant Violet 510^TM^ anti-human CD39 mAb (Biolegend, clone: A1), PE/Cy7 anti-human CD14 mAb (Biolegend, clone:HCD14), APC/Cy7 anti-human CD45 (Biolegend, clone: HI30), Alexa Fluor 488 anti-human Vimentin mAb (BD Biosciences, clone: RV202), PE anti-human Ki-67 mAb (Biolegend, clone: Ki-67), Anti-human Indoleamine2,3-dioxygenase/IDO APC-conjugated mAb (R&D System, Inc., Minneapolis, USA, clone: 700838), CFSE Cell Division Tracker Kit (Biolegend). Additionally, fluorochrome-conjugated antibodies against the following mouse antigens were used for flow cytometry analysis: anti-mouse CD4 fluorescein isothiocyanate (FITC) mAb (eBioscience, clone: GK1.5), FITC anti-mouse Vimentin mAb (BD Biosciences), anti-mouse/rat Foxp3 PE mAb (eBioscience, clone: FJK-16 s), anti-mouse/rat Ki-67 PE mAb (eBioscience, clone: P46013), PE/Cy7 anti-mouse IL-10 mAb (Biolegend, clone: JES5-16E3), PerCP/Cy5.5 anti-mouse CD73 mAb (Biolegend, clone: TY/11.8), APC anti-mouse LAP(TGF-*β*1) mAb (Biolegend, clone: TW7-16B4), Brilliant Violet 421^TM^ anti-mouse CD152 mAb (Biolegend, clone: UC10-4B9), and anti-mouse IDO eFluor® 660 (eBioscience, clone: mIDO-48). Surface staining was performed at 4 °C for 30 min. Intracellular staining was performed using Foxp3 Fix/Perm Buffer Set (4 × , Biolegend) according to the manufacturer protocol. The resulting data were analyzed using the LYSYS II software program (Becton Dickson, Franklin Lakes, NJ, USA).

### The effect of estrogen on the expression of IDO1 in the ectopic lesion

2 × 10^5^ monocytes were co-cultured with ectopic ESCs on 24-well plates (Corning) or alone, adding estrogen (10^−8^ M; Sigma) or not. After 48 h, ESCs and monocytes-derived macrophages from different groups were collected, and then detected the expression of IDO1 by flow cytometry.

### Treatment with estrogen receptor inhibitors in ESCs

Ectopic ESCs were cultured in 24-well plates (Corning) at a density of 1 × 10^5^ cells/well. Pretreated ESCs with estrogen receptor *α* inhibitor (ER_αi_, 10^−6^ M; Sigma), estrogen receptor *β* inhibitor (ER_βi_, 10^−6^ M; Sigma), estrogen receptors inhibitor (ER_i_, 10^−6^ M; Sigma) or not. After 24 h, inhibitors were washed off by PBS (Ji-Nuo, Hangzhou, China), and then added estrogen (10^−8^ M; Sigma) to these groups except control group. After 48 h, the expression of IDO1 in ESCs from different groups was detected by flow cytometry.

### Quantitative real-time polymerase chain reaction

Total RNA from ESCs treated with estrogen (10^−8^ M; Sigma), 1-MT (0.05 mM; Sigma), estrogen (10^−8^ M; Sigma) along with 1-MT (0.05 mM; Sigma), or knockdown of MRC2 with shRNA for 48 h was extracted using the Trizol reagent (Life Technologies, Carlsbad, CA, USA), according to the manufacturer instructions. Total RNA (2 *μ*g) was reverse transcribed into first-stand cDNA (TaKaRa Bio Inc., Japan) following the manufacturer protocol, which was then used as a template for polymerase chain reaction (PCR) amplification. Real-time PCR was performed using ABI PRISM^TM^ 7900 Sequence Detector (Applied Biosystems, Warrington, UK). The primers sequences used are listed in Table 1. PCRs was carried out for 40 cycles using the following conditions: denaturizing at 95 °C for 30 s, annealing at 95 °C for 5 s, and elongation at 60 °C for 34 s. The expression levels of the samples were expressed as arbitrary units defined by the 2^−ΔΔCT^ method. All measurements were performed in triplicate. The specificity of the product was assessed by melting curve analysis.

### Western blot

Protein expression level of MRC2 and *β*-actin were measured by western blot as previously described.^38^ The primary antibodies used were anti-MRC2 mAb (1:1000; Abcam Cambridge, MA, USA), and *β*-actin rabbit mAb (1:1000; Cell Signaling Technology, Inc., Danvers, MA, USA). Horseradish peroxidase-conjugated goat anti-rabbit IgG (1:2000; Arigobio, Taiwan, ROC) was used as the secondary antibody. Quantitative analysis of the relative density of the bands obtained by western blot was performed using Image J (W.S. Rasband, National Institutes of Health, Bethesda, MD, USA).

### shRNA knockdown assay

Ectopic ESCs were cultured on 24-well plates with DMEM/F-12 (Hyclone) plus 10% FBS (Hyclone) in the presence of 100 U/ml penicillin and 100 mg/ml streptomycin, at 37 °C under 5% CO_2_. Lipofectamine 3000 (Invitrogen; Life Technologies, Carlsbad, CA, USA), OPTI-MEM (Gibco BRL, Gaithersburg, MD, USA), and the pGPU6/GFP/Neo-MRC2 short hairpin RNA (shRNA) (GenePharma, Shanghai, China) were used to transfected ESCs according to the manufacturer protocol.

### Mice

We used seven-week-old female C57B/L6 mice (Slaccas Animal Laboratory, Shanghai, China) to establish an endometriosis-disease model as previously described.^39, 40^ Each donor mouse was administered 200 *μ*l estrogen (10^−8^ M; Sigma) via the peritoneal cavity. One week later, donor mice were sacrificed and the uterine horn were collected and minced. Every two recipient mice were then intraperitoneally injected with minced uterine horn tissue from one donor mouse equally. All procedures were performed under aseptic conditions. One week after uterine tissue injection, mice were randomly divided into five groups and each received an intraperitoneal injection of 200 *μ*l PBS (Ji-Nuo), estrogen receptors blockers (10^−6^ M; Sigma), 1-MT (0.05 mM; Sigma), vector (GenePharma, Shanghai, China), or mouse MRC2 shRNA (GenePharma, Shanghai, China) every week. Vector and mouse MRC2 shRNA were mixed with Lipofectamine 3000 (Invitrogen) and OPTI-MEM (Gibco BRL), according to the manufacturer protocol. Two weeks later, we measured the total number and weight of ectopic lesions, the expression of Ki-67 and IDO1 within ectopic lesions, the percentage and the expression of functional molecules of T_reg_ in total ectopic lesions and peritoneal fluid of mice from groups administered PBS, estrogen receptor blockers, 1-MT, vector and MRC2 shRNA (Supplementary Figure 2).

### Statistics

All studies were set up to include three wells per condition, and each experiment was independently repeated more than three times. Data collected from each independent experiment were analyzed using the Graphpad Prism (Graphpad software Inc., La Jolla, CA, USA) statistical package. Paired *t*-test of variance was performed when appropriate. Differences were considered statistically significant at ±S.D.<0.05.

## Figures and Tables

**Table 1:** Characteristics of primers used for qRT-PCR

**Gene**	**Orientation**	**Primer seq**	**Product size**
MRC1	FORWARD	GGAGGATTGTGTGGTGATGA	104
	REVERSE	GACCTTGGCTTCGTGATTTC	
MRC2	FORWARD	TGGGAGAAGACCAAGTGACC	117
	REVERSE	TGTAGATGAGGCTGCTGACG	
CLEC4M	FORWARD	TGGATGGGACTTTCAGACCT	114
	REVERSE	CCCGCTATTGTTGGGTTCT	
PTAFR	FORWARD	CTGGAGTCTGGGATGGTAGC	112
	REVERSE	TCAGCAGGAAATGACACAGC	
GAPDH	FORWARD	AGAAGGCTGGGGCTCATTTG	258
	REVERSE	AGGGGCCATCCACAGTCTTC	
