# Supplementary material for: 1-Methyl-tryptophan attenuates regulatory T cells differentiation due to the inhibition of estrogen-IDO1-MRC2 axis in endometriosis
Source: Cell Death Dis. 2016 Dec 1;7(12):e2489–. doi: 10.1038/cddis.2016.375 (PMC5260991; doi:10.1038/cddis.2016.375)
Supplement: Supplementary Information [file cddis2016375x1.docx]

**Supplementary Information**

**Supplementary Figure 1 Establishment of co-culture systems.** PBMCs were isolated from whole blood and positively sorted CD14^+^ cells were co-cultured with ESCs or not, while negatively sorted naïve CD4^+^ T cells were cultured in 24-well plates coated with 5 μg/ml monoclonal anti-CD3 and 1 μg/ml monoclonal anti-CD28, in the presence of 50 ng/ml recombinant human IL-2. After 48 hours, macrophages and naïve T cells were collected to establish co-culture systems. Differentiation of T_reg_ cells was determined by flow cytometry after five days.

**Supplementary Figure 2 Establishment of endometriosis-disease mouse model.** Seven-week-old female C57B/L6 mice were intraperitoneally injected with estrogen (200 μl, 10^-8^ M). One week later, donor mice were sacrificed and the uterine horn were collected and minced. Every two recipient mice were then intraperitoneally injected with minced uterine horn tissue from one donor mouse equally. All procedures were performed under aseptic conditions. One week after uterine tissue injection, recipient mice were randomly divided into five groups and each received an intraperitoneal injection of 200 μl PBS, estrogen receptors blockers (10^-6^ M), 1-MT (0.05 mM), vector, or mouse MRC2 shRNA every week. Two weeks later, recipient mice were sacrificed, and we measured number and weight of total ectopic lesions, the expression of Ki-67 and IDO1 within ectopic lesions, the percentage and the expression of functional molecules of T_reg_ in total ectopic lesions and peritoneal fluid of mice from groups administered PBS, estrogen receptor blockers, 1-MT, vector and MRC2 shRNA.

**Supplementary Figure 3 Estrogen receptor inhibitor restrains the expression of functional molecules in peritoneal T_reg_ cells *in vivo*.** (**a**) Flow cytometric analysis was used to determine the expression of TGF-β_1_, IL-10, CD73, and CTLA-4 in peritoneal T_reg_ cells *in vivo*. Numbers in quadrants indicate the percentage of cells. (**b-e**) Quantification of TGF-β_1_, IL-10, CD73, and CTLA-4 expression in T_reg_ cells shown in **a**. Values indicate mean ± SD, n=11, **P*<0.05, ***P*<0.01, two-tailed, unpaired t-test.

**Supplementary Figure 4 Schematic of the regulation of estrogen-IDO1-MRC2 axis to the differentiation of T_reg_ cells in endometriosis.** Among peritoneal environment of endometriosis patients, excessive estrogen promotes the expression of IDO1 and MRC2 in ESCs. Therein, high level of IDO1 inhibits the expression of MRC2 in ESCs, which in turn, increases IDO1 and Ki-67 expression. The effect of estrogen on up-regulating MRC2 expression is surpassed by the inhibition of IDO1 on MRC2. Low MRC2 expressed ectopic ESCs may promote monocytes to present stronger signals for Naïve CD4^+^ cells to differentiate into T_reg_ cell and promote T_reg_ suppressive function.

**Table 1 Characteristics of primers used for qRT-PCR.**
