# Supplementary figures and images for: 1-Methyl-tryptophan attenuates regulatory T cells differentiation due to the inhibition of estrogen-IDO1-MRC2 axis in endometriosis
Source: Cell Death Dis. 2016 Dec 1;7(12):e2489–. doi: 10.1038/cddis.2016.375 (PMC5260991; doi:10.1038/cddis.2016.375)

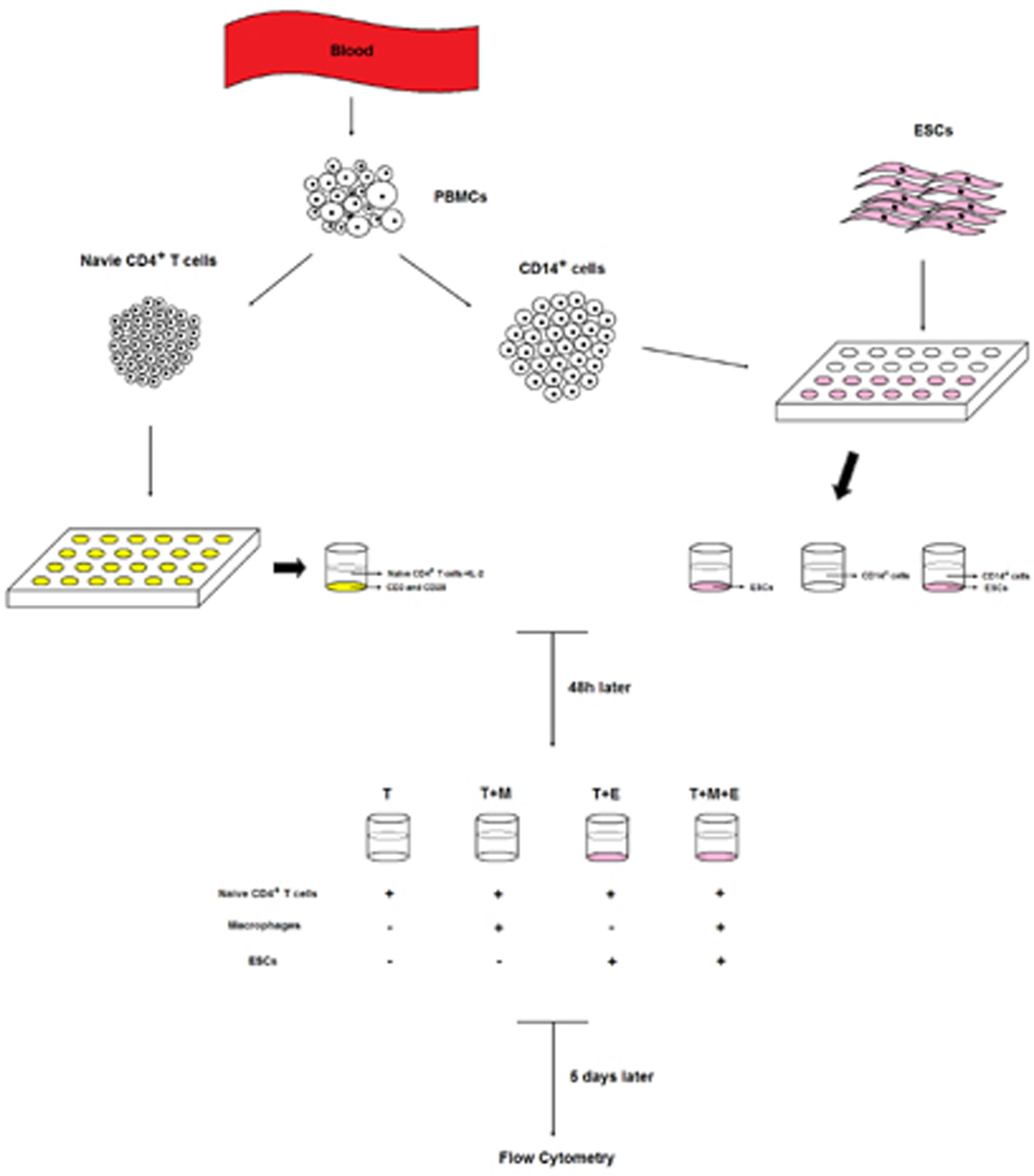

Supplement: Supplementary Figure 1 [file cddis2016375x2.tif]

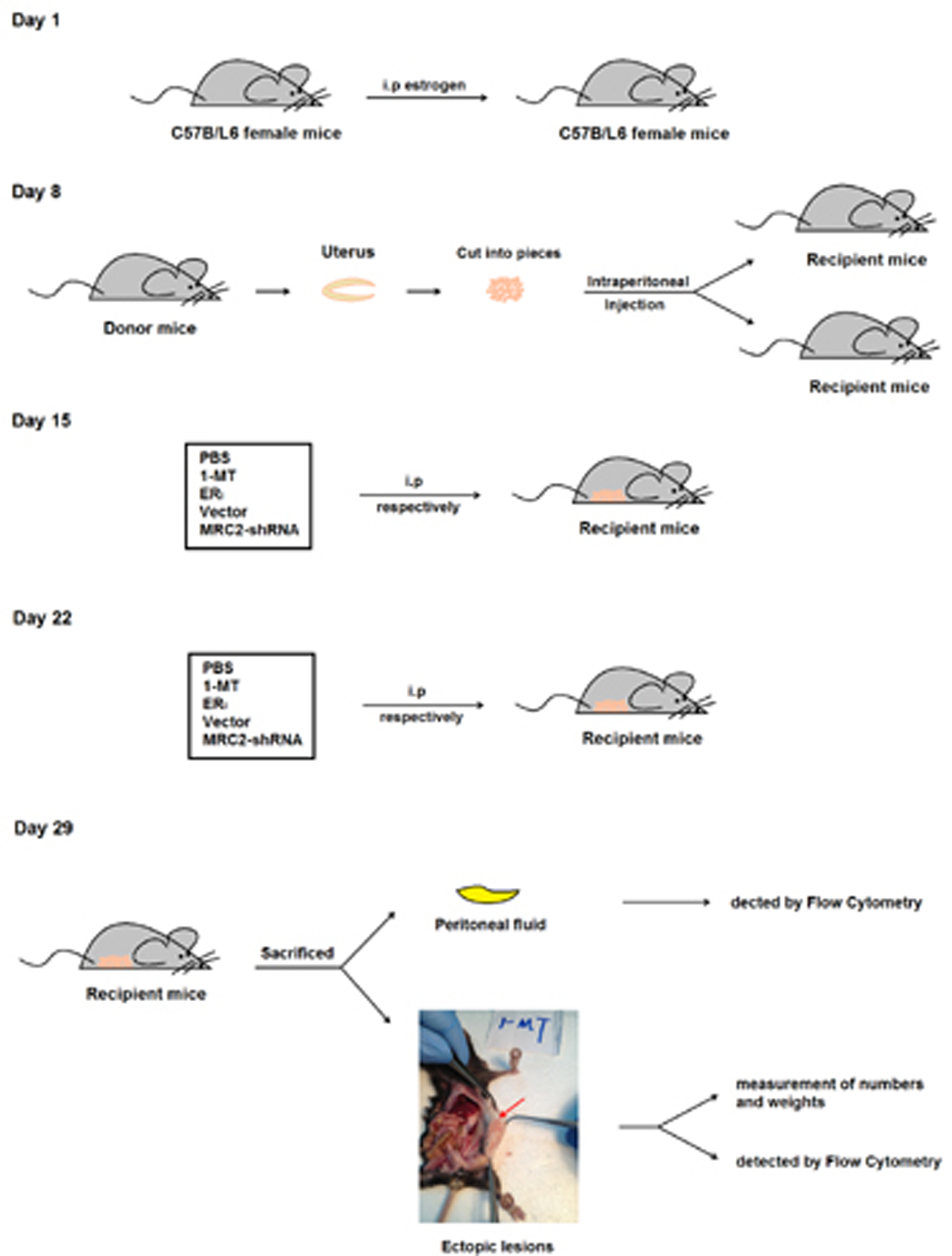

Supplement: Supplementary Figure 2 [file cddis2016375x3.tif]

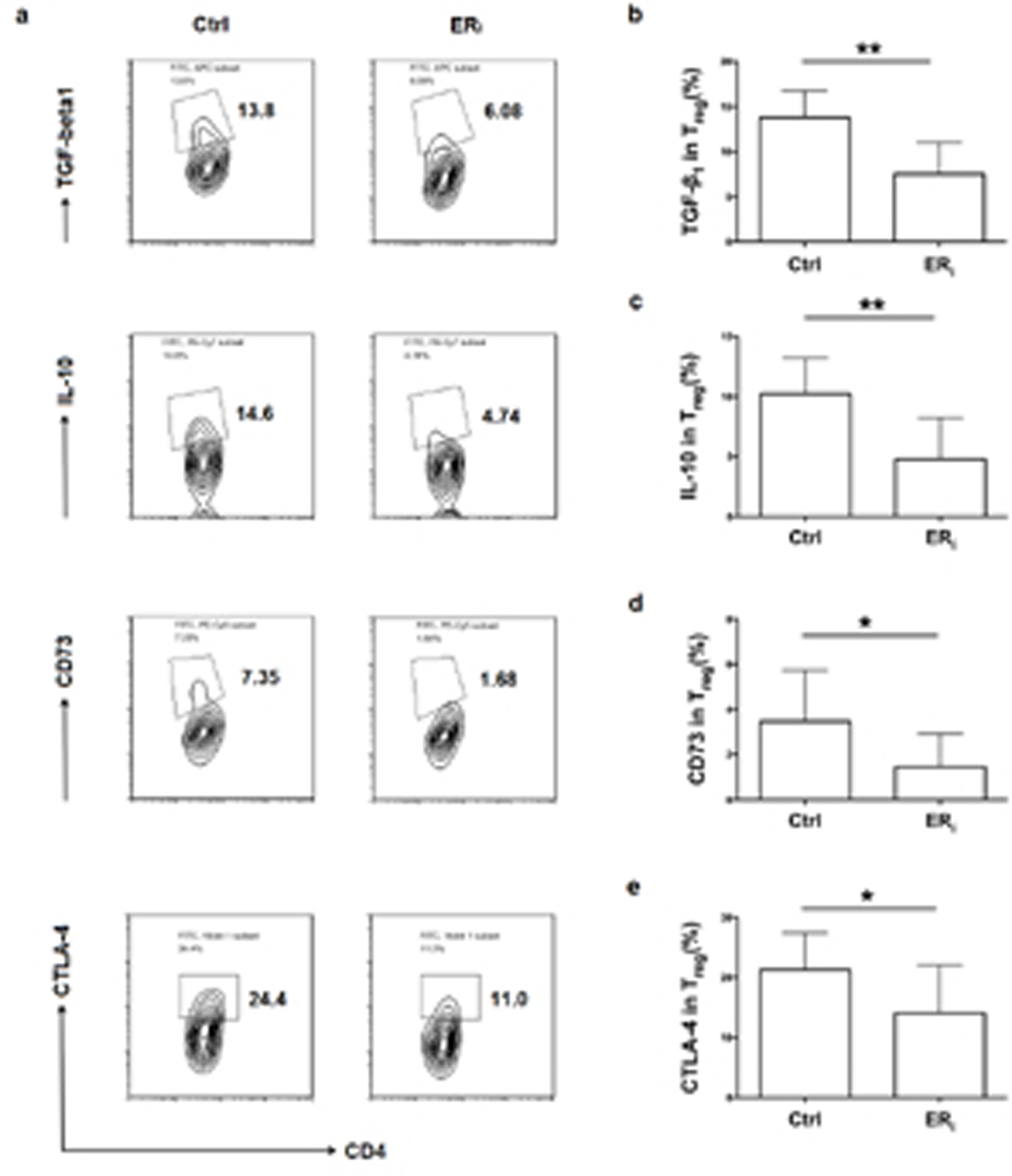

Supplement: Supplementary Figure 3 [file cddis2016375x4.tif]

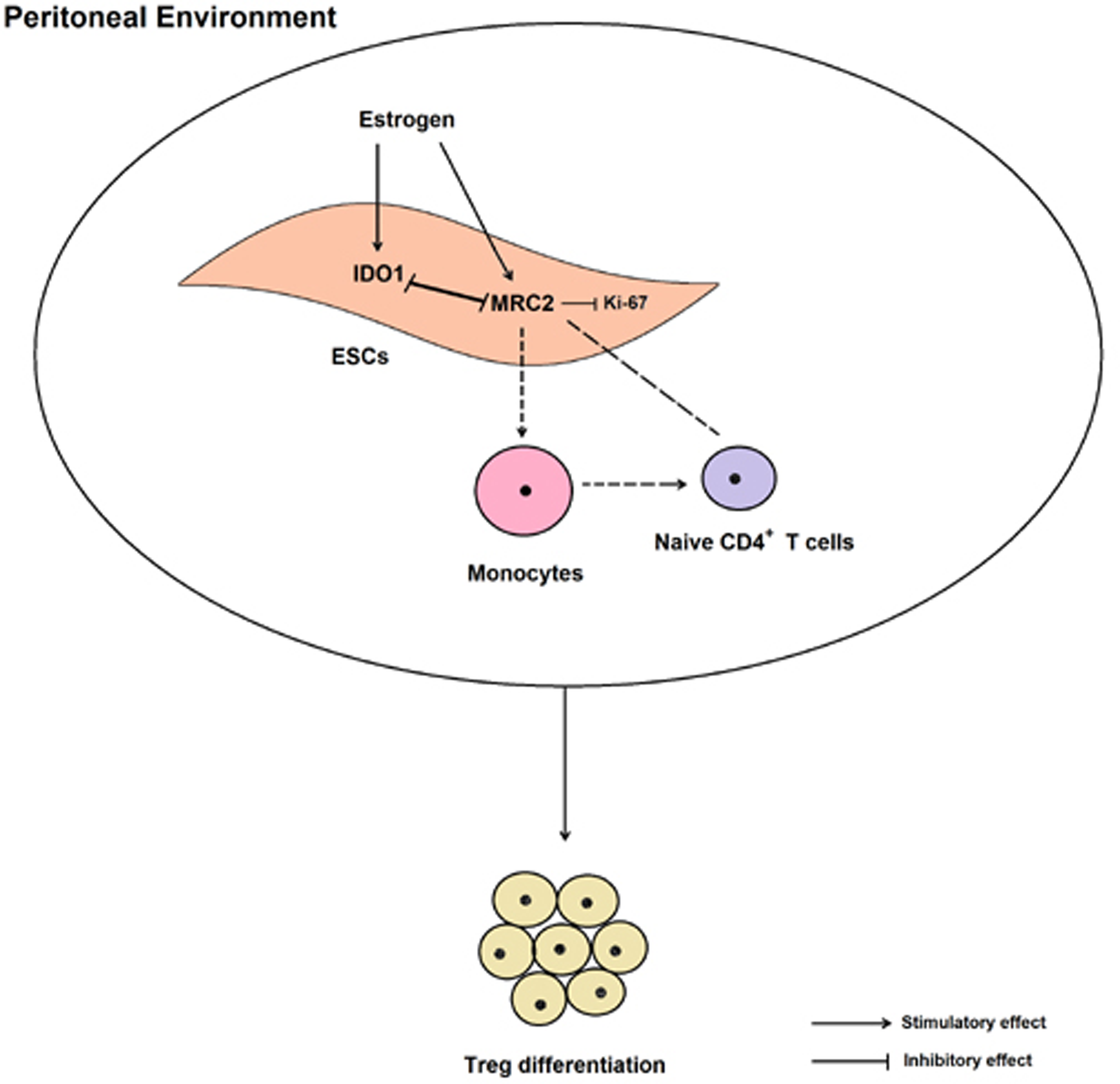

Supplement: Supplementary Figure 4 [file cddis2016375x5.tif]
